# The adenoviral E4orf3/4 is a regulatory polypeptide with cell transforming properties in vitro

**DOI:** 10.1016/j.tvr.2023.200254

**Published:** 2023-01-25

**Authors:** Wing-Hang Ip, Luca D. Bertzbach, Thomas Speiseder, Thomas Dobner

**Affiliations:** Leibniz Institute of Virology (LIV), Department of Viral Transformation, Martinistraße 52, 20251, Hamburg, Germany

**Keywords:** BRK cells, Human adenovirus, HAdV-C5, E4 region, Oncogene, Virus replication

## Abstract

The human adenovirus species C type 5 (HAdV-C5) early region 4 (E4) encodes several distinct polypeptides, defined as E4orf1 to E4orf6/7 according to the order and arrangement of the corresponding open reading frames (ORFs). All E4 gene products operate through a complex network of interactions with key viral and cellular regulatory proteins involved in transcription, apoptosis, cell cycle control, and DNA repair. Here, we generated a set of virus mutants carrying point mutations in the individual E4 genes. The phenotypic characterizations of these mutants revealed that mutations of these ORFs had no or only moderate effects on virus replication. Even a triple mutant that fails to produce E4orf3, E4orf4, and the yet uncharacterized alternatively spliced E4orf3/4 fusion protein, was replicating to wild type levels. The E4orf3/4 protein consists of the N-terminal 33 amino acid residues from E4orf3 and the C-terminal 28 amino acid residues from E4orf4. Intriguingly, we found that, similar to E4orf3, E4orf3/4 possesses properties that support the E1A/E1B-induced transformation of primary rodent cells. These results identify and functionally characterize E4orf3/4 and conclude that E4orf3/4 is another E4 region protein that is dispensable for virus replication but promotes the E1A/E1B-induced transformation of primary rodent cells.

## 1 Introduction

**Fig. 1:**
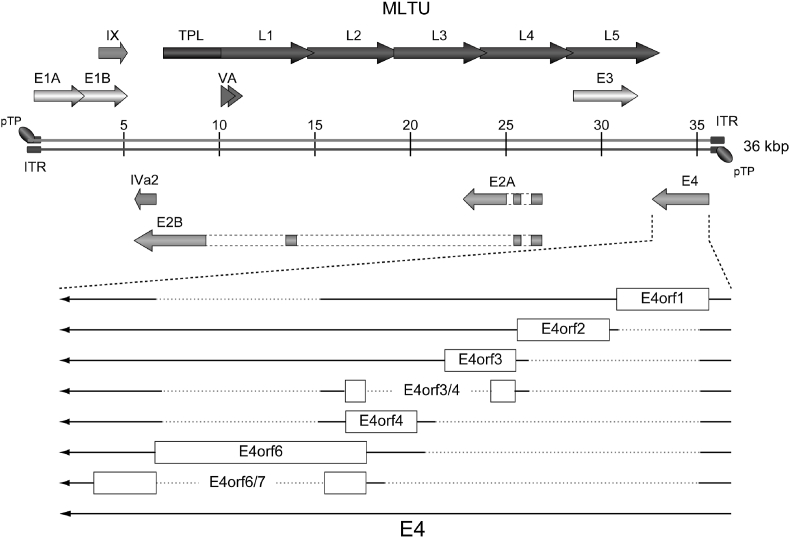
**Schematic representation of the HAdV-C5 E4 region.** Shown is a schematic overview of the HAdV-C5 E4 transcriptional unit with its relative location in the viral genome. Dashed lines indicate introns, solid lines exons, and arrowheads indicate the direction of transcription. A common E4 promoter facilitates the transcription of a primary transcript, which is approximately 2800 nucleotides in length. Up to 18 mRNA species are generated through alternative splicing, which currently encode seven proteins (E4orf1 through E4orf6/7) (modified from Ref. [37]). ITR, inverted terminal repeat; MLTU, major late transcription unit; pTP, precursor terminal protein; TPL, tripartite leader; VA, viral associated RNA.

Human adenoviruses (HAdVs) are intriguing and clinically relevant pathogens that mainly cause ocular, respiratory, and gastrointestinal tract infections [1,2]. HAdV infection can be associated with high morbidities and case-fatality rates in both immunocompetent and immunocompromised patients, and especially in children [[3], [4], [5], [6]]. Human and animal adenoviruses are used as vectors in clinical gene therapy settings and vaccinology for more than three decades and their use as vaccine vectors culminated with the SARS-CoV-2 pandemic [7,8]. One of the most studied HAdVs is HAdV type 5 of species C (HAdV-C5). Its genome comprises a linear 36 kbp dsDNA genome that encodes for more than 30 structural and nonstructural proteins, most of which have been thoroughly characterized. These are clustered into different transcription units (early, intermediate or late) based on their synthesis prior to, during, or following viral DNA replication, respectively [9,10]. The HAdV-C5 early region 4 (E4) encodes at least seven proteins that are designated as E4orf1 through E4orf6/7 according to the arrangement of their open reading frames (ORFs) (Fig. 1). Previous research on the E4 region, however, mainly focused on the proteins encoded by E4orf1, E4orf2, E4orf3, E4orf4, and E4orf6. These E4-encoded proteins act through complex networks of protein-protein interactions with viral and cellular factors involved in transcription, apoptosis, cell cycle control, DNA repair mechanisms, post-translational modifications, and integrity of PML nuclear bodies [11]. Some features are known about the E4orf6/7-encoded protein, which seems to transactivate the cellular transcription factor E2F and play a role in viral transformation [[12], [13], [14], [15]].

According to northern blot and cDNA sequence analyses, the predicted E4orf3/4 protein is a 61 amino acid fusion protein consisting of the 33 amino-terminal residues of E4orf3 and the 28 carboxy-terminal residues of E4orf4 [16]. However, some previous research on E4 mRNA profiles showed that no E4orf3/4 mRNA was detected [17,18], possibly due to its rather low abundance in infected cells [16].

In this report, we set to assess the consequences of E4 region gene mutations, with an emphasis on the E4orf3/4 protein, on viral DNA replication. Moreover, we aimed at deciphering the role of E4orf3/4 in adenovirus-induced cell transformation.

Our study shows that E4orf3/4 is nonessential for virus replication. However, we discovered that E4orf3/4, like E4orf3, enhances adenoviral E1A and E1B oncoprotein-mediated transformation of primary baby rat kidney (pBRK) cells.

## 2 Materials and methods

### 2.1 Cells and culture conditions

A549 cells (ACC 107; German Collection of Microorganisms and Cell Cultures, Braunschweig, Germany) and pBRK cells [19] were maintained in Dulbecco's Modified Eagle Medium (DMEM; Gibco; Carlsbad, CA, USA) which was supplemented with 10% fetal calf serum (PAN Biotech; Aidenbach, Germany) and 1% penicillin/streptomycin (10,000 U/ml penicillin; 10 mg/ml streptomycin in 0.9% NaCl, PAN Biotech) at 37 °C and 5% CO_2_. Cell cultures were regularly monitored for the absence of mycoplasma contamination.

### 2.2 Plasmids and transient transfections

All plasmids are described and referenced in Table 1. For transient transfections, subconfluent cells were treated with a mixture of DNA and 25 kDa linear polyethylenimine (PEI) as described [20] and were harvested/analyzed at different time points post-transfection.

**Table 1:** Plasmids.

Plasmid	Vector	Transgene	Reference
pE1A	pML	HAdV-C5 E1A	[38]
pE1B	pcDNA3	HAdV-C5 E1B–55K	[38]
pE4orf3	pcDNA3	HAdV-C5 E4orf3	[30]
pE4orf3/4	pcDNA3	HAdV-C5 E4orf3/4	This work

### 2.3 Viruses

All virus mutants (Table 2 and Table S1) were generated from the HAdV-C5 wild type (wt) reference strain H5*pg*4100 exactly as described earlier [21,22] and all previously unpublished primers we used are listed in Table S2. Each mutation that we generated in H5*pg*4100 was confirmed by Sanger sequencing. Moreover, we sequenced essential viral genes to exclude possible off-target mutations.

**Table 2:** Viruses.

Virus	Phenotype			Reference
	E4orf3	E4orf3/4	E4orf4	
HAdV-C5 wt (H5*pg*4100)	+	+	+	[21]
HAdV-C5 E4orf3- (H5*pm*4150)	–	+	+	[39]
HAdV-C5 E4orf3/4- (H5*pm*4163)	+	–	+	This work
HAdV-C5 E4orf4- (H5*pm*4166)	+	+	–	[25]
HAdV-C5 E4orf3- 4–3/4- (H5*pm*4234)	–	–	–	This work

### 2.4 qPCRs

To determine viral DNA production, A549 cells were harvested at 1, 24, 48, and 72 h post-infection (h p.i.) and analyzed by qPCR. The QIAamp DNA Mini Kit (Qiagen; Hilden, Germany) was used to isolate DNA from infected cells at different time points p.i. at a multiplicity of infection (MOI) of 1 following the manufacturer's protocol. Quantitative PCRs (qPCRs) were performed on the DNA samples using a Rotor-Gene 6000 (Corbett Life Sciences; Sydney, Australia), SYBR Green chemistry, and primers specific for E1B (forward primer 5’-GAC AGG GCC TCT CAG ATG CT-3’; reverse primer 5’-TGG CTA CGT GAA TGG TCT TCA G-3’) as well as 18S (forward primer 5’-CGG CTA CCA CAT CCA AGG AA-3’; reverse primer 5’-GCT GGA ATT ACC GCG GCT-3’).

### 2.5 Protein analysis

Cell pellets were lysed in RIPA buffer (50 mM Tris-HCl/pH 8.0, 150 mM NaCl, 5 mM EDTA/pH 8.0, 0.1% SDS, 1% NP-40, 0.5% sodiumdeoxycholate) supplemented with protease inhibitors (PMSF 1 mM, aprotinin 10 U/ml, leupeptin 1 μg/ml, pepstatin 1 μg/ml und DTT 1% (v/v)) on ice for 30 min. Lysates were sonicated and subsequently centrifuged to pellet the cell debris (11,000 rpm, 3 min, 4 °C). The protein concentrations were determined photometrically using Bradford Reagent (BioRad; Hercules, CA, USA). Protein samples were boiled for 5 min at 95 °C in Laemmli buffer before they were loaded on SDS gels where equal amounts of protein lysate were then separated by SDS-polyacrylamide gel electrophoresis (SDS-PAGE). For immunoblotting, the samples were then transferred to nitrocellulose membranes, incubated, and visualized as described previously [23] using the antibodies that are listed in Table 3.

**Table 3:** Antibodies.

Antibody	Company or reference
α-E1A (M73)	[40]
α-E1B–55K (2A6)	[41]
α-E2A (B6-11)	[42]
α-E4orf3 (6A11)	[30]
α-E4orf4 (2419)	[43]
α-E4orf6 (1807)	[44]
α-L4-100K (6B10)	[45]
α-late (L133)	[46]
α-β-actin (AC-15)	Sigma Aldrich
HRP α-mouse IgG	GE Healthcare
HRP α-rat IgG	GE Healthcare
HRP α-rabbit IgG	GE Healthcare

### 2.6 Transformation assays

Transformation assays were performed as described [19,24] with minor adjustments to the transfection procedure. Briefly, pBRK cells were seeded in 6-well plates and transfected with the respective plasmids by PEI transfection (Table 1). Transfected cells were cultivated for approximately 21 days with weekly media changes until multilayered cell accumulations (foci) were visible. These foci were then visualized by crystal violet staining and quantified.

### 2.7 Statistical analyses

All statistical analyses were performed using GraphPad Prism v9 (GraphPad Software; San Diego, CA, USA). Specific information on the statistical tests is provided in the respective figure legends. Data were considered significantly different if the p-value was ≤0.05.

## 3 Results and discussion

### 3.1 E4orf3/4 is dispensable for viral DNA replication

**Fig. 2:**
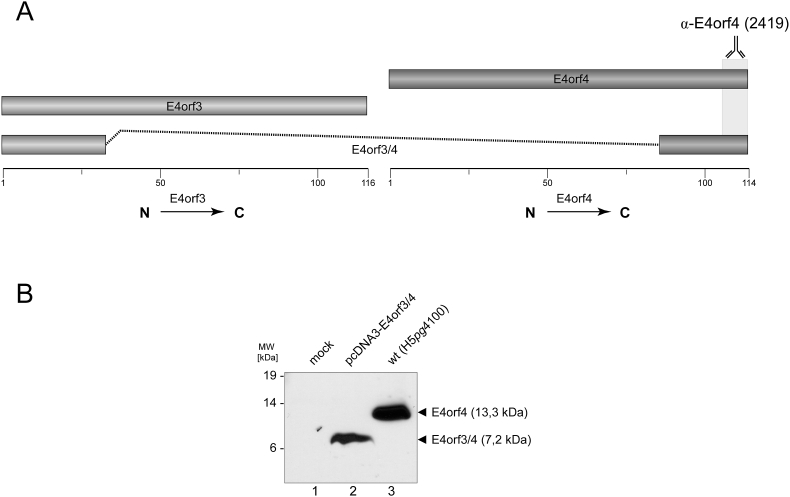
**Detection of E4orf3/4 and E4orf4.** (A) Antibody binding region of α-E4orf4 (2419) to E4orf4 and E4orf3/4. (B) A549 cells were transfected with 5 μg pcDNA3-E4orf3/4 (Table 1) or infected with HAdV-C5 wt (MOI 50). At 48 h p.t. or 48 h p.i., cells were harvested and whole cell protein lysates were detected by Western blotting using the α-E4orf4 monoclonal antibody 2419. We loaded equal amounts of total protein (150 μg) into all lanes. Molecular weights (MW) in kilodaltons (kDa) are indicated on the left, while corresponding proteins as well as their molecular weights are indicated on the right.

First, we used an E4orf4-specific antibody to detect E4orf3/4 in transiently transfected cells for the first time. In wt-infected cells, however, we detected only E4orf4 (Fig. 2). Interestingly, previous research on E4 mRNA profiles showed that no E4orf3/4 mRNA could be detected [17,18]. This could be due to (i) the use of deletion mutants instead of splice-site mutations which may distort the splicing pattern [17], (ii) investigations of HAdV-C2 instead of -C5 [18], or (iii) merely because of the apparent low abundance of E4orf3/4 in infected cells – which we believe is the most likely answer for these observed results [16,17].

**Fig. 3:**
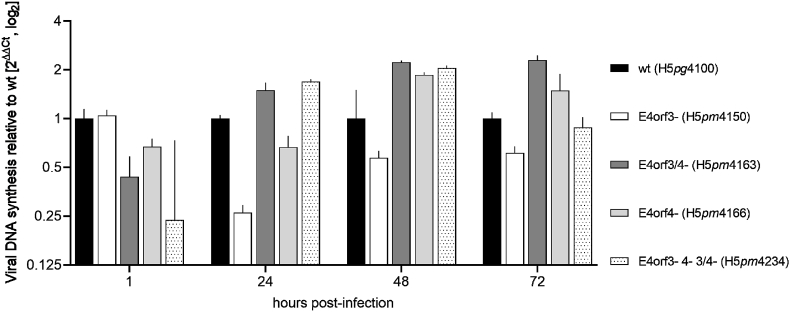
**Virus replication.** A549 cells were infected with H5pg4100 and the different mutants (as indicated, MOI 1). Total DNA was isolated at 1–72 h p.i. (as indicated) and quantified by qPCR using primers for E1B and 18S (as a housekeeping gene for normalization). The experiment was done in two triplicate measurements and error bars indicate standard deviations.

We then set to determine whether the different mutations in the E4 region affect viral DNA replication using the E4orf3/4-mutants as well as other E4 region mutants as essential controls. For this purpose, we infected A549 cells with our set of E4 region mutants and isolated DNA from infected cells at different time points post-infection. Next, we analyzed the DNA samples and thus, adenoviral DNA replication, by qPCR (Fig. 3).

We found that virus replication in A549 cells was only marginally affected in 72 h time course infections with HAdV-C5 wt (H5*pg*4100), the mutants E4orf3- (H5*pm*4150), E4orf3/4- (H5*pm*4163), E4orf4- (H5*pm*4166), as well as the triple mutant E4orf3- 4–3/4- (H5*pm*4234) (Fig. 3). This result is consistent with observations in which E4orf3-or E4orf4-negative virus mutants show no major replication defect compared to their parental strains [[25], [26], [27]]. For additional characterization and validation of the introduced single mutations on the protein level, and to further examine their effects on viral replication and viral protein synthesis, we assessed the expression of E4 region proteins as well as other selected early and late HAdV genes over time (Figs. S1, S2, and S3). From these experiments, we again confirmed the mutations of our null mutants, as these proteins were not expressed in the respective infections (Fig. S1). The expression of selected early and late HAdV genes over time confirmed that the viral life cycle is only marginally, if at all, affected by the introduced mutations (Figs. S2 and S3).

### 3.2 E4orf3/4 exhibits cell-transforming properties

To shed light on functional properties of E4orf3/4 and taking into consideration the fact that other E4-encoded proteins also possess cell-transforming properties, we set to analyze the cell-transforming properties of E4orf3/4 together with E1A and E1B–55K. For that, we quantified the transformation of pBRK cells using classical transformation assays [19] in which the cells were transfected with one or more recombinant plasmids encoding proteins from the E1 or E4 region. In these assays, the transformed cells form multilayered colonies (foci) during an incubation period of 21 days, while non-transformed cells undergo cell death [19]. The number of foci is an indication of the growth stimulating capabilities and thus, transforming potential of viral gene products in this in vitro system. Together with E1A, E1B–55K is capable of completely transforming non-permissive cells in vitro [28,29]. Combined with the E1A and E1B–55K, it has also been shown that E4orf3 possesses cell-transforming potential [30], whereas E4orf4 exhibits pro-apoptotic activity [31,32] making it unsuitable to be used in transformation experiments.

**Fig. 4:**
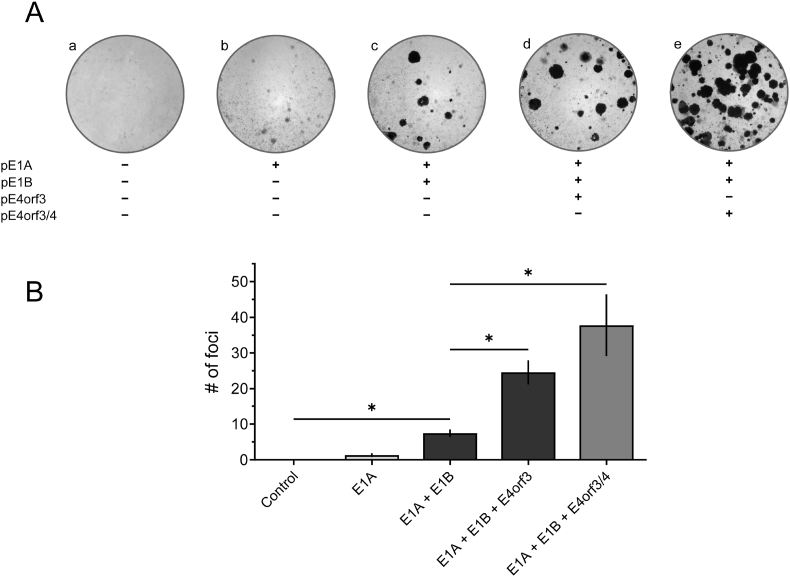
**Transforming potential of E4orf3 and E4orf3/4 in cooperation with E1A and E1B–55K.** (A) Primary BRK cells were transfected with the recombinant plasmids pE1A, pE1B, pE4orf3, or pE4orf3/4, cultured for 3–4 weeks, and stained with crystal violet. The depicted plates show one representative experiment of three independent assays, each time repeated in triplicate. (B) Foci of each plate were quantified and plotted and shown are the mean values with standard deviations from three independent experiments. Asterisks indicate significant differences (p-values were obtained from a one-way ANOVA with a Dunnett's multiple comparisons test (*p ≤ 0.05), comparing the data to ‘E1A + E1B’).

Consistent with previously performed analyses, we show that E1A alone immortalizes pBRK cells, indicated by a few single-layered cell colonies that we detected as bright foci by crystal violet staining (Fig. 4A, image b). In contrast, when E1A and E1B–55K were co-transfected, pBRK cells underwent a complete transformation (Fig. 4A, image c) [33]. Moreover, and as previously reported, E4orf3 enhances the transforming ability of E1A and E1B–55K (Fig. 4A, image d) [30]. Remarkably, we show that co-transfecting E1A and E1B–55K with E4orf3/4 increases focus formation about fivefold compared with E1A plus E1B–55K alone, while co-transfecting E1A, E1B–55K, and E4orf3 tripled the number of foci (Fig. 4A, image e, and Fig. 4B). This is the first evidence for a profound transformation-promoting potential of E4orf3/4. Despite the sequence homologies of E4orf3/4 with E4orf3 as well as with E4orf4, our work suggests that this spliced viral (fusion) protein might lack the E4orf4-mediated pro-apoptotic functions, but the transformation-promoting properties of E4orf3 are additionally enhanced in E4orf3/4. Similar to E4orf3, we speculate that Eorf3/4 is incapable to transform on its own [30]. We could not detect E4orf3/4 in transformed BRKs at later time points, where only E1A and E1B were present (data not shown). Furthermore, we could not establish cell lines (neither of human nor of rat origin) via plasmid transfection that stably express E4orf3/4 and we thus hypothesize that E4orf3/4 possesses transformation-promoting functions that are important very early in the transformation process. At the same time, E4orf3/4 is not required to maintain the transformed status of the cells.

## 4 Conclusions

In this report, we present our investigations on the hitherto uncharacterized E4orf3/4 gene product. The primary goal of these investigations was to elucidate the role of the E4orf3/4 protein in adenovirus-infected cells in lytic infection and to determine its cell-transforming properties through transformation assays. First, the present results confirm that the E4 region of human adenoviruses encodes the mRNA of the fusion protein of parts of open reading frames 3 and 4, E4orf3/4, which can be isolated, cloned and overexpressed to produce the E4orf3/4 protein in transfection experiments. Second, our characterizations of several E4 virus mutants demonstrate that E4orf3/4 is dispensable for virus replication. Third, and analogous to E4orf3, we provide evidence that E4orf3/4 possesses growth-stimulating properties, as the viral protein increases the focus formation of primary rat kidney cells in cooperation with the adenoviral E1A and E1B–55K oncoproteins. Intriguingly, we show that E4orf3/4 significantly increases the efficiency of foci-formation, even compared to E4orf3. Thus, E4orf3/4, together with E4orf3 and E4orf6, is the third protein of the E4 region that possesses transformation-promoting properties. Quite recent HAdV RNAseq data provided insight into splicing events, viral mRNA biogenesis, and alternative splicing isoforms in the complex HAdV transcriptome by nanopore direct RNA sequencing [[34], [35], [36]]. According to these studies, E4orf3/4 mRNA expression mostly only reaches levels several logs below those of E4orf3 and E4orf4. Future experiments will determine if this is due to mRNA stability and/or accelerated mRNA degradation or if that even could be a cell-dependent effect. It should be noted that the presented data are primarily based on in vitro overexpression experiments. Whether and to what extent the E4orf3/4 protein is expressed in viral infections as well as its roles in in vivo infections, however, remain to be determined.

## Funding

The Leibniz Institute of Virology is supported by the Freie und Hansestadt Hamburg and the 10.13039/501100003107German Bundesministerium für Gesundheit (BMG).

## Author contribution statement

Wing-Hang Ip: formal analysis, investigation, writing – review and editing, visualization.

Luca D. Bertzbach: formal analysis, writing – original draft, writing – review and editing, visualization.

Thomas Speiseder: conceptualization, methodology, formal analysis, investigation, writing – review and editing, visualization.

Thomas Dobner: conceptualization, methodology, formal analysis, writing – review and editing, project administration, funding acquisition, supervision.

## Declaration of competing interest

The authors declare that they have no known competing financial interests or personal relationships that could have appeared to influence the work reported in this paper.

## Data Availability

No data was used for the research described in the article.
