# Supplementary material for: The adenoviral E4orf3/4 is a regulatory polypeptide with cell transforming properties in vitro
Source: Tumour Virus Res. 2023 Jan 25;15:200254. doi: 10.1016/j.tvr.2023.200254 (PMC10258410; doi:10.1016/j.tvr.2023.200254)
Supplement: Multimedia component 1 [file mmc1.pdf]

Supplementary information for:

The adenoviral E4orf3/4 is a regulatory polypeptide with cell transforming properties in vitro

by Ip *et al*.

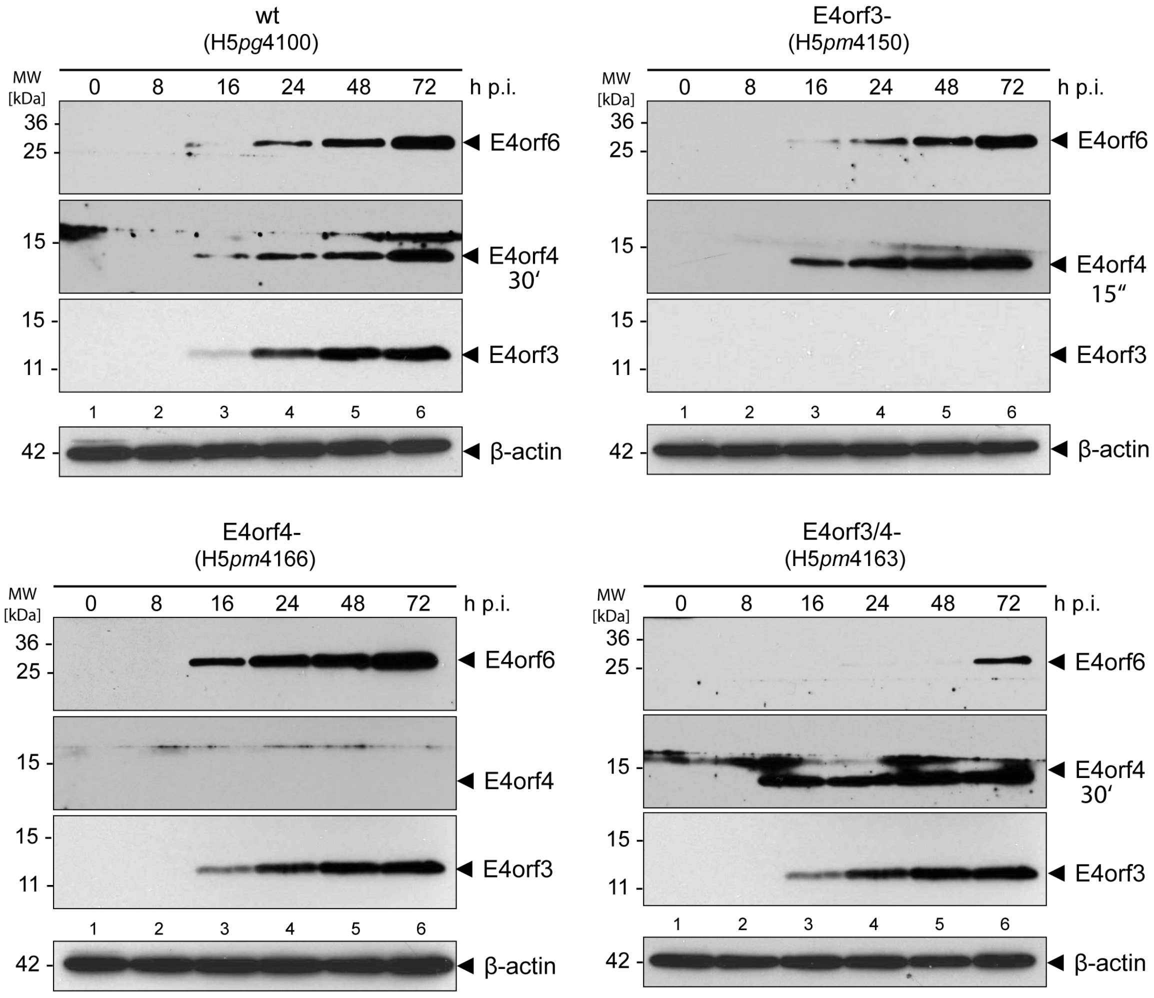

Figure S1: Steady-state levels of E4-encoded adenoviral proteins. A549 cells were infected with the indicated HAdV-C5 E4 virus mutants (MOI 5) and harvested at the indicated time points. Equal amounts of whole cell extracts were separated through 15% SDS-polyacrylamide gels, transferred to PVDF membranes, and visualized by immunoblotting. Detection was performed using the primary antibodies directed against E4orf3, E4orf4, E4orf6, β-actin and corresponding HRP-conjugated secondary antibodies (Tab. 3). Molecular weights (MW) in kilodaltons (kDa) are indicated on the left, while corresponding proteins are indicated on the right. Mock is indicated as 0 h p.i.

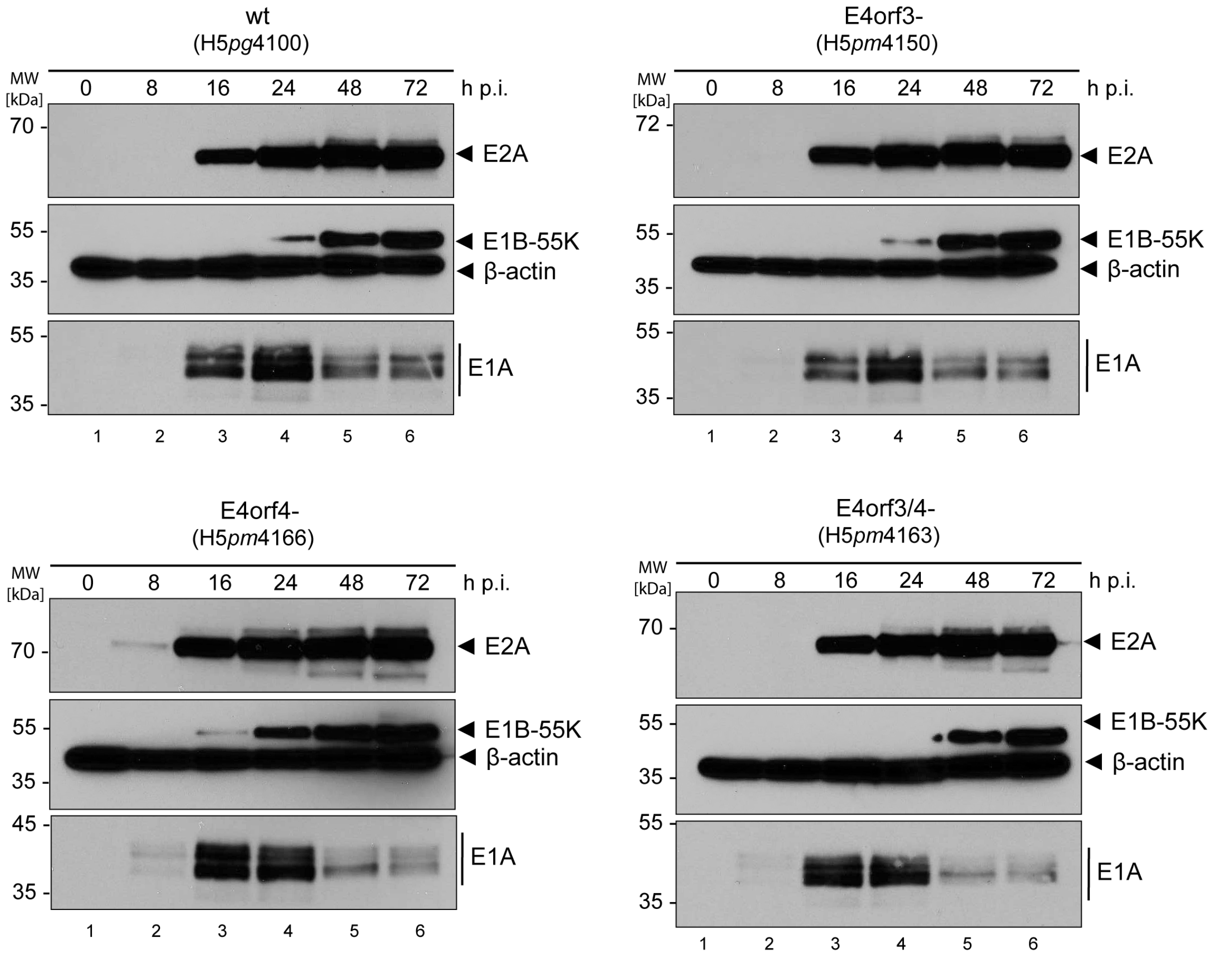

Figure S2: Steady-state levels of early adenoviral proteins. A549 cells were infected with the indicated HAdV-C5 E4 virus mutants (MOI 5) and harvested at the indicated time points. Equal amounts of whole cell extracts were separated through 15% SDS-polyacrylamide gels, transferred to PVDF membranes, and visualized by immunoblotting. Detection was performed using the primary antibodies directed against E2A, E1A, E1B-55K, β-actin and corresponding HRP-conjugated secondary antibodies (Tab. 3). Molecular weights (MW) in kilodaltons (kDa) are indicated on the left, while corresponding proteins are indicated on the right. Mock is indicated as 0 h p.i.

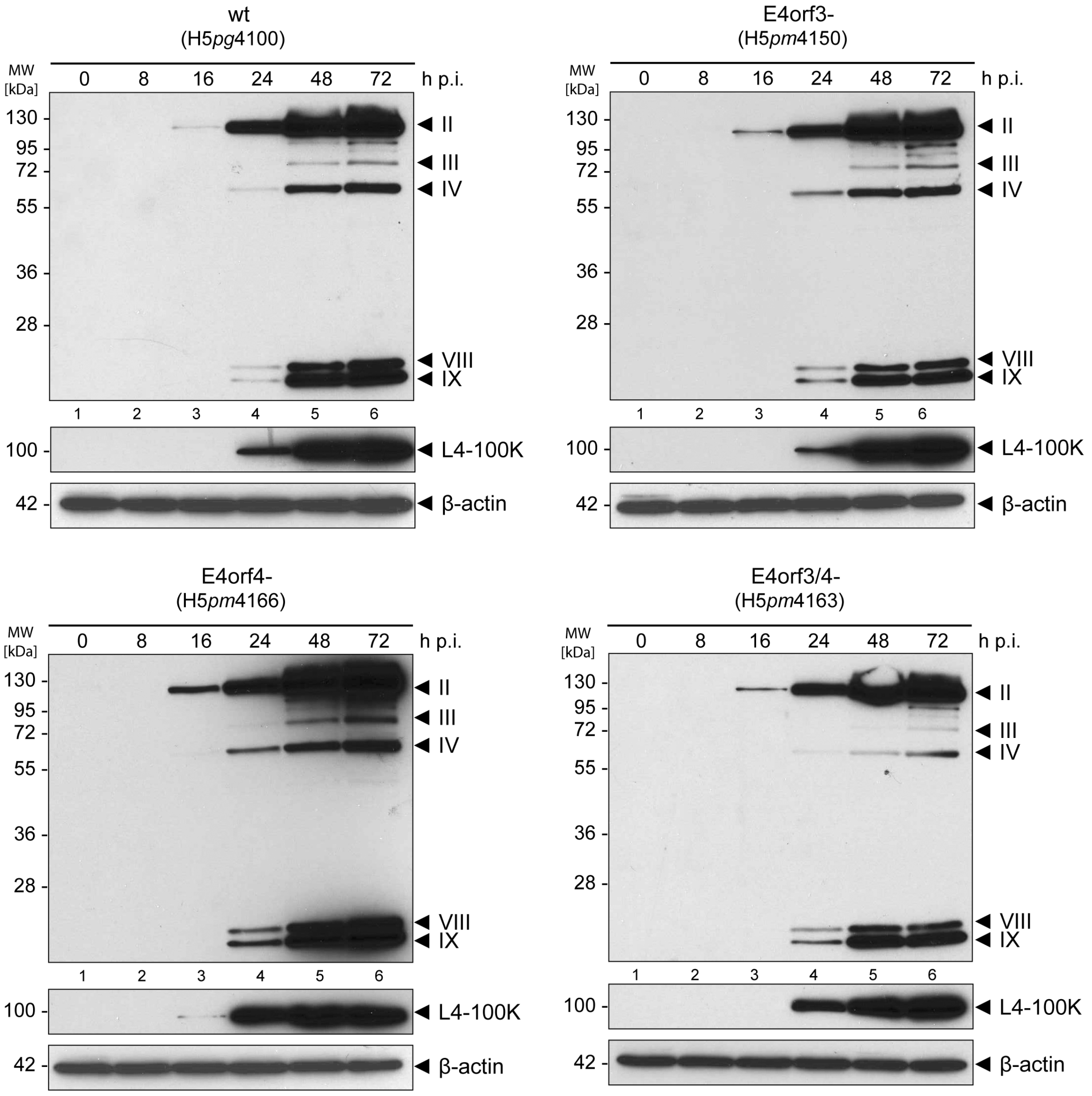

Figure S3: Steady-state levels of late adenoviral proteins. A549 cells were infected with the indicated HAdV-C5 E4 virus mutants (MOI 5) and harvested at the indicated time points. Equal amounts of whole cell extracts were separated through 12% SDS-polyacrylamide gels, transferred to nitrocellulose membranes, and visualized by immunoblotting. Detection was performed using the primary antibodies directed against late adenoviral proteins (polyclonal serum), L4-100K, β-actin and corresponding HRP-conjugated secondary antibodies (Tab. 3). Capsid proteins: II, hexon; III, penton, IV, fiber; VIII and IX, hexon-associated proteins. Molecular weights (MW) in kilodaltons (kDa) are indicated on the left, while corresponding proteins are indicated on the right. Mock is indicated as 0 h.p.i.

Table S1: Additional details on virus mutants.

Virus	Characteristics
H5*pm*4163	HAdV-C5 E4orf3/4 null mutant harboring a mutation of the splice donor site D2a [Dix and Leppard, 1993 (JVI)].
H5*pm*4234	HAdV-C5 E4orf3- 4- 3/4- triple null mutant; a combination of H5*pm*4150, H5*pm*4163, and H5*pm*4166.

Table S2: Previously unpublished mutagenesis primers.

Primer #	Orientation	Sequence (5' – 3')	Used for…
E4orf3/4 NoDo	forward	GAG ATA TAT TGA GAC GCT GGC GAG ATG AG	…the orf3/4 mutation
	reverse	CTC ATC TCG CCA GCG TCT CAA TAT ATC TC	
E4orf3^{-}	forward	GGT GGC GAG ATT GAG AAT TAT TTG GGC	…the orf3 mutation
	reverse	GCC CAA ATA ATT CTC AAT CTC GCC ACC	
